# 1.5–2 cm tumor size was not associated with distant metastasis and mortality in small thyroid cancer: A population-based study

**DOI:** 10.1038/srep46298

**Published:** 2017-04-11

**Authors:** Kyunghwa Han, Eun-Kyung Kim, Jin Young Kwak

**Affiliations:** 1 Department of Radiology and Research Institute of Radiological Science, Severance Hospital, Yonsei University College of Medicine, 50 Yonsei-ro, Seodaemun-gu, Seoul 03722, South Korea

## Abstract

Most guidelines for the diagnosis and management of thyroid nodules have suggested fine-needle aspiration as a diagnostic tool, with some of these previously published guidelines suggesting a cutoff size of 1.5 cm. In thyroid cancers (1–2 cm), we hypothesized that tumors 1.5 cm or larger had more unfavorable clinical outcomes than ones smaller than 1.5 cm nodules. Using the Surveillance, Epidemiology, and End Results database, we identified 14,117 patients diagnosed with only primary thyroid cancer between 1988 and 2007. After multivariable adjustment, we found that having a tumor 1.5 cm or larger in size was not associated with distant metastasis [adjusted odds ratio, 1.18; 95% confidence interval (CI), 0.95 to 1.48; *P* = 0.14] or the two causes of death (adjusted subdistributional hazard ratio (SDHR), 1.40; 95% CI, 0.96 to 2.04; *P* = 0.08 for thyroid cancer mortality; adjusted SDHR, 1.06; 95% CI, 0.88 to 1.27; *P* = 0.55 for noncancer mortality). Using a population-based cohort, in patients with primary thyroid cancer with a tumor size of 1.5–2 cm, there was no increased association with distant metastasis or probability of death, when compared with patients with primary thyroid cancer with a tumor size of 1.0–1.5 cm.

With the increased use of imaging studies, especially high-resolution ultrasonography (US), the number of thyroid nodules detected has rapidly increased^1^,^2^. While it is crucial not to miss cancers among these detected thyroid nodules, it is also critically important to detect and diagnose cancers which may be clinically significant. Fine-needle aspiration (FNA) is the standard method used to diagnose thyroid nodules detected on US, and it is known to be both accurate and cost-effective^3^,^4^. Although there have been several guidelines that recommend FNA for thyroid nodules^3^,^5^,^6^,^7^,^8^,^9^, a worldwide consensus on indications for FNA of thyroid nodules has not yet been achieved.

Because distant metastases can occur in patients with thyroid cancers larger than 2 cm, all nodules larger than 2 cm except simple cysts are recommended for FNA even if they have very few suspicious US features^3^,^6^,^8^,^10^. Also, most guidelines do not recommend FNA for thyroid nodules smaller than 1 cm except in high risk patients because most papillary microcarcinomas without unfavorable features have no clear negative outcomes^11^,^12^. For thyroid nodules 1–2 cm in size, most guidelines suggest FNA based on the size and US features of the thyroid nodule^3^,^5^,^6^,^8^. The Society of Radiologists in Ultrasound (SRU), the National Comprehensive Cancer Network, and the American Thyroid Association use a size cutoff of 1.5 cm according to how suspicious the thyroid nodules appear on US;^3^,^5^,^6^,^8^ however, the guidelines of the American Association of Clinical Endocrinologists/Associazione Medici Endocrinologi/European Thyroid Association do not consider a size cutoff of 1.5 cm^9^. Using a 1.5 cm size cutoff along with US features as an indication for FNA can be burdensome in real practice. Taking into account that guidelines should be easily applicable in clinical practice, a less complicated subclassification system/algorithm is required to indicate which nodules need FNA. Recently, the diagnostic performances and abilities of six major worldwide guidelines to identify aggressive cancers were compared for patients with small thyroid nodules^13^. Although there were no significant associations between positive findings that indicated FNA and distant metastasis, recurrence or persistence, the study power of this previous study was too low to detect significant differences. With a population-based cohort, we sought to find an association with distant metastasis or thyroid cancer mortality using a size cutoff of 1.5 cm in patients with small thyroid cancers between 1 cm and 2 cm. We hypothesized that among thyroid nodules 1 cm or larger but smaller than 2 cm, nodules 1.5 cm or larger had more unfavorable clinical outcomes than ones smaller than 1.5 cm. We thought that our research could help provide potential evidence to support the 1.5 cm size cutoff as an indication for FNA.

## Results

### Patient Characteristics

Table 1 lists patient demographics and clinical characteristics according to tumor size. The significant factors between the tumor size groups (group 1: 1–1.4 cm, group 2: 1.5–1.9 cm) were as follows; histologic subtype, extrathyroidal extension, cervical lymph node metastasis, distant metastasis, and radiation therapy. The number of patients 55 years or older for group 1 and group 2 were 1653 (22.2%) and 1395 (20.9%), respectively. The rates of extrathyroidal extension, cervical lymph node metastasis and distant metastasis in group 2 were 17.1%, 64.9% and 2.7%, respectively.

The cumulative incidence over the entire follow-up time was significantly different between the tumor size subgroups among patients who died of thyroid cancer-related causes (*P* = 0.02), but was not different among patients who died of noncancer causes (*P* = 0.85).

The most frequent causes of noncancer mortality were heart disease (31.2%), other causes (18.3%), cerebrovascular diseases (8.1%), and chronic obstructive pulmonary disease (6.3%). The median follow-up time in group 1 and group 2 was 105 months (range, 0 to 299 months) and 110 months (range, 0 to 299 months), respectively.

### Distant Metastasis

At univariable analysis, group 2 was associated with increased risk of distant metastasis compared to group 1 (OR, 1.65; 95% CI, 1.08 to 1.68; *P* = 0.008). Other significant risk factors for distant metastasis were male sex, other thyroid carcinoma except papillary or follicular carcinomas, extrathyroidal extension, cervical lymph node metastasis, surgery type (no surgery, total thyroidectomy *v* lobectomy), and radiation therapy. Multivariable analysis showed that group 2 was not significantly associated with the risk of distant metastasis compared to group 1 after adjustment for patient demographics and clinical characteristics (adjusted OR, 1.19; 95% CI, 0.95 to 1.49; *P* = 0.13) (Table 2).

We also performed subgroup analyses according to an age cutoff of 55 years to identify groups associated with distant metastasis (Table 3). In univariable analysis, group 2 was significantly associated with distant metastasis when patients were younger than 55 years (OR, 1.39; 95% CI, 1.08 to 1.78; *P* = 0.01), but was not significantly associated with distant metastasis when patients were 55 years or older (OR, 1.22; 95% CI, 0.77 to 1.93; *P* = 0.39). Multivariable analyses showed that group 2 was not significantly associated with distant metastasis for both age groups, for both patients 55 years or older (adjusted OR, 0.88; 95% CI, 0.54 to 1.45; *P* = 0.62) and patients younger than 55 years (adjusted OR, 1.27; 95% CI, 0.99 to 1.63; *P* = 0.06), after adjustment for patient and tumor characteristics.

### Competing Risk Analysis

**Figure 1:**
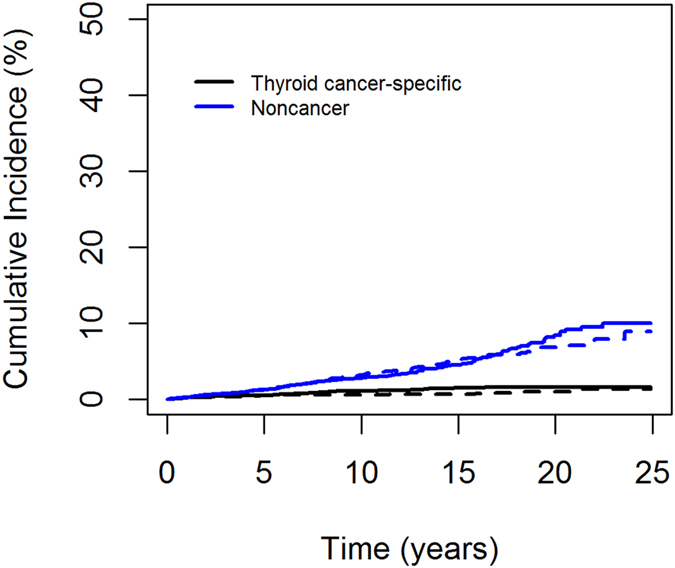
Cumulative incidences of competing causes of death in patients with thyroid cancer according to tumor size. The black line indicates patients who died from thyroid cancer, and the blue line indicates patients who died from noncancer causes. Solid line, 1.5–1.9 cm; Dashed line, 1–1.4 cm.

The cumulative incidence curves are shown in Fig. 1. For thyroid cancer-specific mortality, the 5-year cumulative incidences of death for group 1 and group 2 were 0.51% and 0.57%, the 10-year cumulative incidences were 0.64% and 1.16%, the 15-year cumulative incidences were 0.69% and 1.51%, and the 20-year cumulative incidences were 1.07% and 1.61%, respectively. For noncancer deaths, the 5-year cumulative incidences of death for group 1 and group 2 were 1.34% and 1.26%, the 10-year cumulative incidences were 3.23% and 2.86%, the 15-year cumulative incidences were 5.16% and 4.53%, and the 20-year cumulative incidences were 6.87% and 8.46%, respectively.

On univariable analysis, the risk of thyroid cancer-related mortality was significantly increased in group 2 than in group 1, whereas the risk of noncancer mortality was not significantly associated with both groups (Table 4). In patients younger than 55 years, group 2 was not significantly associated with thyroid cancer-specific mortality, or noncancer mortality, but it was significantly associated with increased thyroid cancer-specific mortality for patients 55 years or older (Table 3).

On multivariable analysis, group 2 did not significantly increase thyroid cancer-related mortality or noncancer mortality (Table 5). Increased thyroid cancer-specific mortality was associated with patient age 55 years or older, male sex, cancers other than papillary carcinoma, extrathyroidal extension, cervical lymph node metastasis, distant metastasis, and no surgery. Significant risk factors for noncancer mortality were patient age 55 years or older, male sex, black race, no surgery, and radiation therapy (Table 5). Group 2 was not significantly associated with death resulting from thyroid cancer or noncancer causes on multivariable analyses in both age subgroups (Table 3).

## Discussion

We found that a tumor size of 1.5 cm or larger in small thyroid nodules was not associated with death resulting from thyroid cancer or other noncancer causes and was not associated with distant metastasis on multivariable analyses using logistic regression and the subdistributional hazards models. These findings were consistent when patients were stratified according to age, with the newly updated cutoff of 55 years in the cancer staging system^14^. Significant differences for age disappeared between the two tumor size groups when a cutoff age of 55 years was applied rather than the traditional cutoff age of 45 years, and other results were not notably different (data not shown).

In this study, noncancer causes of death were treated as competing risk rather than censoring or all-cause mortality, because deaths due to causes unrelated to thyroid cancer interfere with the ability to observe deaths resulting from thyroid cancer. The effects of the covariates on multivariable models differ for each cause of death. Therefore, we treated deaths from noncancer causes as competing risk events. Another study using the SEER database found that patients with thyroid cancer who did not undergo surgical treatment were observed with a smaller 5-year overall survival rate, older age, advanced cancer stage, and more distant metastases; however, this study did not explain the reason for these differences because the SEER database does not offer information about comorbidities^15^. Our analysis incorporated competing risks using the COD variable in the SEER database instead of comorbidity information to infer the effects of noncancer causes of death. The results of our multivariable analysis suggest that patients who do not undergo surgery are at significantly high risk of noncancer mortality. The most common cause of noncancer mortality in our study was heart disease. Non-surgical patients might not be eligible for surgery because of their comorbidities, and this in turn, affected the occurrence of distant metastases and mortalities. We resolved these issues by the multivariable competing risks model which included non-surgical patients.

Thyroid cancer is the most common cancer among endocrine tumors and its incidence is increasing rapidly throughout the world^16^,^17^,^18^. The main cause of this increase is the rising number of incidentally detected thyroid nodules at imagings with US, computed tomography, and magnetic resonance imaging. Considering that most thyroid nodules are benign, it is important to identify and select nodules for which FNA is necessary. Most societies recommend FNA for nodules 1 cm or larger which also have the respective US features and for nodules 2 cm or larger except for those with a simple cyst^3^,^6^,^7^,^8^. Since the SRU recommended FNA for thyroid nodules, a size cutoff of 1.5 cm has been used to decide whether or not to perform FNA in accordance to each society’s US criteria^3^. The cutoff tumor size of 1.5 cm currently suggested by thyroid nodule management guidelines would be acceptable if cancers 1.5 cm or larger showed poorer clinical outcomes compared to tumors smaller than 1.5 cm. Some studies were cited in guidelines for FNA of thyroid nodules to support a size cutoff of 1.5 cm. A long-term study of 1,355 patients with thyroid cancers demonstrated that tumors smaller than 1.5 cm had lower 30-year recurrence and lower cancer mortality rates than tumors between 1.5 cm and 4.4 cm, and 4.5 cm or larger, respectively^19^. However, this study included cancers from 0.2 cm to 10 cm in size; thus, there was insufficient evidence to support using the 1.5 cm cutoff to indicate FNA for nodules 1–2 cm in size, as was suggested by previous guidelines. Although multivariable analyses with adjustment for patient and tumor characteristics were performed, tumor size was considered as continuous scale. Another study using the SEER database found that the 10-year relative survival rates for tumors sized 1.5 cm or larger and tumors less than 1.5 cm were 95.4% and 99.8%, respectively^20^. However, this study included thyroid cancers which were less than 5 mm to thyroid cancers 4 cm or larger; moreover, no adjustment was made for tumor and treatment characteristics.

Developing appropriate criteria to select which small thyroid nodules should undergo FNA is essential to prevent the risk of missing clinically significant cancers, to decrease the number of patients who undergo unnecessary FNAs, and to enhance user convenience. Established methodological standards and researches for developing guidelines have suggested that guideline features should be of high quality, be trustworthy, and be implementable^21^,^22^,^23^,^24^. Therefore, it is very surprising that there has been no evidence that proves that thyroid nodules 1.5 cm or larger have poorer clinical outcomes compared to nodule smaller than 1.5 cm when focusing on nodules 1 cm or larger but smaller than 2 cm. So far, a worldwide consensus on indications for FNA of thyroid nodules has not been achieved, making the need for logical evidence more crucial. Our study is the first population-based study to demonstrate that there is not enough evidence to support a cutoff tumor size of 1.5 cm for managing thyroid nodules after adjusting for patient, tumor, and treatment characteristics although this study cannot provide direct evidence for some guidelines which use a cutoff tumor size of 1.5 cm to indicate FNA for thyroid nodules.

This study has several strengths. By using the COD variable in the SEER database, we were able to classify specific causes of mortality, and were able to perform analyses with an adequate number of deaths occurring in thyroid cancer patients. Hence, the SEER database allows us to perform multivariable competing risk analyses adjusting for patient and tumor characteristics, and surgical treatments. The SEER data gave our study, a population-based long-term follow-up study, adequate statistical power that could not have been achieved by single institution studies. Also, results from a population-based SEER study have more generalizability and reliability.

Our study does have several limitations. The SEER database does not include detailed data on comorbidity, disease recurrence, and sonographic features which have been included in most FNA guidelines. External validations using well-constructed prospective multi-institutional data for thyroid nodules may provide relevant validity for various guidelines. Also, the SEER database might have some coding errors. Before constructing the study population, we first excluded patients who were classified as having thyroid cancer but recorded as having non-thyroid malignancy as the histological subtype. Information regarding COD in the SEER database is acquired from a patient’s death certificate, and there is a chance that this information might be misclassified. However, a previous study has found the COD coding in SEER to be highly accurate^25^.

In summary, we found that having a tumor 1.5 cm or larger in size was not associated with distant metastasis and the two causes of death (thyroid cancer mortality and noncancer mortality) after adjustment for patient, tumor, and clinical characteristics. Therefore, further studies using the 1.5 cm cutoff as an indication for FNA in thyroid nodules are needed.

## Methods

### SEER Database and Study Population

The Surveillance, Epidemiology, and End Results (SEER) database covers approximately 30% of the United States population^26^. We contacted the SEER program of the National Cancer Institute in August 2015. Our study only selected cases diagnosed after 1988 because information on extent of disease such as lymph node involvement, or detailed information on tumor size was required. 127512 patients with thyroid cancer (C73.9) as the first primary malignancy as well as histologically diagnosed tumors of the thyroid were identified according to the International Classification of Diseases for Oncology, 3^rd^ Edition (ICD-O-3).

To include a minimum of 5 years of follow-up survival data, we only selected patients who were diagnosed up to 2007. Tumors with sizes equal to or larger than 2 cm or smaller than 1 cm were excluded. We excluded patients who were diagnosed with other cancers than thyroid cancer. Patients with no available information on race, surgical findings (extrathyroidal extension, lymph node involvement, surgery type), and radiation therapy were also excluded. Patients with a cause of death (COD) record that indicated an unavailable death certificate or patients with no records of survival time were excluded. Patients with this data but with no COD recorded (SEER code of 41000) were excluded as well. The final study cohort was comprised of 14117 patients with thyroid cancers.

Variables included age at diagnosis (55 years or older vs younger than 55 years), sex, race, extrathyroidal extension, cervical lymph node metastasis, distant metastasis, surgery type, and radiotherapy. The presence of distant metastasis was determined using the SEER historical staging system. Extent of surgery was grouped as no surgery, lobectomy, and total thyroidectomy including subtotal or near-total thyroidectomy.

Thyroid cancer-specific death was ascertained with the SEER COD record of 32010, and noncancer mortality was also defined. Survival time was determined from the SEER records on survival time, that is, from date of diagnosis to date of last contact. The date of last contact was as follows: for deaths, date of death; for survivors, the date of last contact if before December 31, 2012; or December 31, 2012, if date of last contact was after 2012.

Institutional review board approval was not required because data from the SEER program is publically available and anonymized.

### Statistical Analyses

We divided the patients into two groups, group 1 (nodules 1 cm or larger and smaller than 1.5 cm) and group 2 (nodules 1.5 cm or larger and smaller than 2 cm). Patient demographics and clinical characteristics were compared between the two groups using Pearson χ^2^ test or Fisher’s exact test.

Univariable and multivariable logistic regression models were used to examine the effects of patient demographics and clinical characteristics for the presence of distant metastasis. Odds ratios (ORs) and 95% confidence intervals (CIs) were estimated.

Cumulative incidence curves for death in the presence of the competing risk were estimated and compared between the two groups by Gray’s method^27^. We used proportional subdistribution hazards regression modeling proposed by Fine and Gray to estimate the effects of the covariates on the cumulative probability of specific causes of death^28^. The proportional hazards assumption was assessed graphically with plots of Schoenfeld-type residuals against time for each cause of death in the model and by testing the time-dependent covariate for each variable.

The latest American Joint Committee on Cancer (AJCC) staging criteria for differentiated thyroid cancer consider patient age at diagnosis, with 55 years of age being the cutoff value for staging^14^. Reflecting this guideline, we performed separate analyses for both distant metastasis and mortality according to age subgroups using 55 years as the cutoff.

All tests were two-sided, and a *P* value of less than.05 was considered statistically significant. Statistical analyses were performed using SAS (version 9.2; SAS Institute Inc, Cary, NC) and R statistical software (version 3.1.1; R Foundation, Vienna, Austria). The R package cmprsk was used for competing risks analyses^29^.

## Additional Information

**How to cite this article:** Han, K. *et al*. 1.5-2 cm tumor size was not associated with distant metastasis and mortality in small thyroid cancer: A population-based study. *Sci. Rep.* **7**, 46298; doi: 10.1038/srep46298 (2017).

**Publisher's note:** Springer Nature remains neutral with regard to jurisdictional claims in published maps and institutional affiliations.

## Figures and Tables

**Table 1:** Patient Demographics and Clinical Characteristics.

Characteristics	1–1.4 cm (n = 7442)	1.5–1.9 cm (n = 6675)	*P* Value
No. (%)	No. (%)		
Age at diagnosis			
≥55 years	1653 (22.2)	1395 (20.9)	0.06
<55 years	5789 (77.8)	5280 (79.1)	
Male sex	1314 (17.7)	1198 (18.0)	0.65
Race			0.94
Black	324 (4.3)	286 (4.3)	
White	6238 (83.8)	5610 (84.0)	
Other	880 (11.8)	779 (11.7)	
Histologic subtype			<0.001
Other	199 (2.7)	248 (3.7)	
Follicular	191 (2.6)	320 (4.8)	
Papillary	7052 (94.8)	6107 (91.5)	
Extrathyroidal extension			0.003
Absent	6308 (84.8)	5533 (82.9)	
Present	1134 (15.2)	1142 (17.1)	
Cervical lymph node metastasis			<0.001
Absent	2930 (39.4)	2345 (35.1)	
Present	4512 (60.6)	4330 (64.9)	
Distant metastasis			0.008
Absent	7291 (98.0)	6494 (97.3)	
Present	151 (2.0)	181 (2.7)	
Surgery type			0.27
Lobectomy	991 (13.3)	828 (12.4)	
No surgery	46 (0.6)	41 (0.6)	
Total thyroidectomy	6405 (86.1)	5806 (87.0)	
Radiation therapy			<0.001
None	3105 (41.7)	2511 (37.6)	
Yes	4337 (58.3)	4164 (62.4)	
Death resulting from thyroid cancer	51 (0.7)	73 (1.1)	0.02^a^
Death resulting from noncancer cause	245 (3.3)	229 (3.4)	0.85^a^

^a^*P* value from Gray’s test.

**Table 2:** Univariable and Multivariable Analyses for Distant Metastasis (N = 14117).

Characteristic	Univariable		Multivariable	
OR (95% CI)	*P* Value	OR (95% CI)	*P* Value	
Tumor size (1.5–1.9 cm vs 1–1.4 cm)	1.65 (1.08 to 1.68)	0.008	1.19 (0.95 to 1.49)	0.13
Age (≥55 years vs < 55 years)	1.06 (0.82 to 1.38)	0.65	1.05 (0.80 to 1.38)	0.72
Male sex	1.91 (1.50 to 2.44)	<0.001	1.62 (1.26 to 2.08)	<0.001
Race				
Black	0.56 (0.27 to 1.16)	0.12	0.85 (0.41 to 1.78)	0.67
White	0.91 (0.66 to 1.26)	0.57	1.07 (0.77 to 1.49)	0.70
Other	Ref. 1		Ref. 1	
Histologic subtype				
Other	2.11 (1.34 to 3.31)	0.001	2.37 (1.46 to 3.84)	<0.001
Follicular	0.85 (0.45 to 1.61)	0.62	1.25 (0.65 to 2.40)	0.50
Papillary	Ref. 1		Ref. 1	
Extrathyroidal extension (presence vs absence)	5.89 (4.73 to 7.35)	<0.001	4.62 (3.68 to 5.80)	<0.001
Cervical lymph node metastasis (presence vs absence)	5.38 (3.76 to 7.68)	<0.001	4.49 (3.12 to 6.44)	<0.001
Surgery type				
Lobectomy	Ref. 1		Ref. 1	
No surgery	4.13 (1.39 to 12.29)	0.01	5.30 (1.64 to 17.18)	0.006
Total thyroidectomy	2.21 (1.42 to 3.45)	<0.001	1.52 (0.96 to 2.42)	0.07
Radiation therapy (presence vs absence)	2.47 (1.90 to 3.22)	<0.001	1.98 (1.50 to 2.62)	<0.001

OR, odds ratio; CI, confidence interval.

**Table 3:** Summary of Relative Risks of Tumor Size (1.5–1.9 cm vs 1–1.4 cm) for Distant Metastasis and Competing Causes of Death According to Age Subgroups (N = 14117).

Subgroup	Distant metastasis		Thyroid cancer-specific mortality		Noncancer mortality	
OR (95% CI)	*P* Value	SDHR (95% CI)	*P* Value	SDHR (95% CI)	*P* Value	
Univariable analysis						
Age < 55 years	1.39 (1.08 to 1.78)	0.01	1.34 (0.68 to 2.6)	0.40	0.87 (0.64 to 1.19)	0.39
Age ≥ 55 years	1.22 (0.77 to 1.93)	0.39	1.75 (1.15 to 2.66)	0.01	1.12 (0.90 to 1.39)	0.33
Multivariable analysis^a^						
Age < 55 years	1.27 (0.99 to 1.63)	0.06	1.09 (0.53 to 2.22)	0.82	0.87 (0.64 to 1.18)	0.37
Age ≥ 55 years	0.88 (0.54 to 1.45)	0.62	1.47 (0.93 to 2.31)	0.10	1.14 (0.91 to 1.42)	0.27

OR, odds ratio; CI, confidence interval; SDHR, subdistribution hazard ratio.

^a^Adjusted for sex, race, histologic subtype, extrathyroidal extension, cervical lymph node metastasis, distant metastasis, surgery type, and radiotherapy.

**Table 4:** Univariable Analyses of Factors Associated with Causes of Death (N = 14117).

Characteristic	Thyroid cancer-specific mortality		Noncancer mortality	
SDHR (95% CI)	*P* Value	SDHR (95% CI)	*P* Value	
Tumor size (1.5–1.9 cm vs 1–1.4 cm)	1.55 (1.09 to 2.22)	0.02	0.98 (0.82 to 1.18)	0.85
Age ( ≥ 55 years vs < 55 years)	10.6 (7.18 to 15.8)	<0.001	9.16 (7.56 to 11.1)	<0.001
Male sex	3.42 (2.40 to 4.89)	<0.001	1.97 (1.62 to 2.41)	<0.001
Race				
Black	1.15 (0.44 to 2.96)	0.78	2.74 (1.8 to 4.17)	<0.001
White	0.99 (0.57 to 1.70)	0.96	1.26 (0.93 to 1.71)	<0.001
Other	Ref. 1		Ref. 1	
Histologic subtype				
Other	6.82 (4.25 to 10.94)	<0.001	1.70 (1.12 to 2.6)	0.01
Follicular	1.82 (0.84 to 3.93)	0.13	1.17 (0.75 to 1.81)	0.49
Papillary	Ref. 1		Ref. 1	
Extrathyroidal extension (presence vs absence)	8.23 (5.75 to 11.80)	<0.001	1.15 (0.9 to 1.45)	0.26
Cervical lymph node metastasis (presence vs absence)	2.16 (1.34 to 3.5)	<0.001	0.98 (0.78 to 1.24)	0.87
Distant metastasis (presence vs absence)	16.21 (10.9 to 24.09)	<0.001	1.51 (0.95 to 2.4)	0.08
Surgery type				
Lobectomy	Ref. 1		Ref. 1	
No surgery	7.32 (2.38 to 22.52)	<0.001	8.10 (4.92 to 13.32)	<0.001
Total thyroidectomy	1.29 (0.74 to 2.24)	0.37	0.66 (0.53 to 0.83)	<0.001
Radiation therapy (presence vs absence)	1.34 (0.93 to 1.95)	0.12	0.61 (0.51 to 0.73)	<0.001

SDHR, subdistribution hazard ratio; CI, confidence interval.

**Table 5:** Multivariable Analyses of Factors Associated with Causes of Death (N = 14117).

Characteristic	Thyroid cancer-specific mortality		Noncancer mortality	
SDHR (95% CI)	*P* Value	SDHR (95% CI)	*P* Value	
Tumor size (1.5–1.9 cm vs 1–1.4 cm)	1.33 (0.91 to 1.96)	0.14	1.04 (0.87 to 1.25)	0.68
Age (≥55 years vs < 55 years)	8.77 (5.69 to 13.51)	<0.001	8.65 (7.09 to 10.56)	<0.001
Male sex	2.14 (1.45 to 3.15)	<0.001	1.65 (1.33 to 2.03)	<0.001
Race				
Black	1.49 (0.59 to 3.79)	0.40	2.59 (1.69 to 3.99)	<0.001
White	1.13 (0.63 to 2.04)	0.68	1.27 (0.94 to 1.73)	0.12
Other	Ref. 1		Ref. 1	
Histologic subtype				
Other	4.8 (2.62 to 8.8)	<0.001	0.85 (0.51 to 1.42)	0.53
Follicular	2.16 (1.05 to 4.45)	0.036	1.05 (0.67 to 1.62)	0.84
Papillary	Ref. 1		Ref. 1	
Extrathyroidal extension (presence vs absence)	5.22 (3.27 to 8.31)	<0.001	1.03 (0.8 to 1.32)	0.84
Cervical lymph node metastasis (presence vs absence)	1.96 (1.19 to 3.24)	0.008	1.32 (1.04 to 1.68)	.021
Distant metastasis (presence vs absence)	6.14 (3.67 to 10.28)	<0.001	1.16 (0.65 to 2.06)	0.62
Surgery type				
Lobectomy	Ref. 1		Ref. 1	
No surgery	5.18 (1.87 to 14.36)	0.002	6.19 (3.58 to 10.7)	<0.001
Total thyroidectomy	1.08 (0.56 to 2.05)	0.83	0.84 (0.66 to 1.07)	0.16
Radiation therapy (presence vs absence)	0.98 (0.62 to 1.53)	0.91	0.77 (0.63 to 0.93)	0.008

SDHR, subdistribution hazard ratio; CI, confidence interval.
